# The History of Clotting Factor Concentrates Pharmacokinetics

**DOI:** 10.3390/jcm6030035

**Published:** 2017-03-20

**Authors:** Massimo Morfini

**Affiliations:** Past President of Italian Association of Haemophilia Centres (AICE), Via dello Statuto n.1, I-50129 Firenze, Italy; massimo.morfini@unifi.it; Tel.: +39-348-230-6928

**Keywords:** clotting factor concentrates, pharmacokinetics, compartment methods, Non-Compartment Analysis, Bayesian compromise, PopPK, prophylaxis, In Vivo Recovery, Clearance, Half-life, Volume of distribution

## Abstract

Clotting factor concentrates (CFCs) underwent tremendous modifications during the last forty years. Plasma-derived concentrates made the replacement therapy feasible not only in the hospital but also at patients’ home by on-demand or prophylactic regimen. Virucidal methods, implemented soon after hepatitis and AIDS outbreak, and purification by Mabs made the plasma-derived concentrates safer and purer. CFCs were considered equivalent to the other drugs and general rules and methods of pharmacokinetics (PK) were applied to their study. After the first attempts by graphical methods and calculation of In Vivo Recovery, compartment and non-compartment methods were applied also to the study of PK of CFCs. The bioequivalence of the new concentrates produced by means of recombinant DNA biotechnology was evaluated in head-to-head PK studies. Since the beginning, the large inter-patient variability of dose/response of replacement therapy was realized. PK allowed tailoring haemophilia therapy and PK driven prophylaxis resulted more cost effective. Unfortunately, the need of several blood samples and logistic difficulties made the PK studies very demanding. Recently, population PK (PopPK) has been applied to the prediction of CFCs dosing by Bayesian methodology. By PopPK also sparse data may allow evaluating the appropriateness of replacement therapy.

## 1. Introduction

The absorption and elimination of drugs attracted the attention of scientists and physicians since the beginning of modern medicine, but the pharmacokinetics developed during the second half of last century [1]. The advent of personal computers allowed for sophisticated and iterative procedures for best fitting and modelling. Pharmacokinetics (PK) deals with the Adsorption, Distribution, Elimination and Metabolism (ADEM) of drugs into the recipient’s body. The in vivo behaviour and decay of drugs depends on the body anatomical and functional characteristics of recipients. On the contrary, pharmacodynamics studies the effects of drug on the body of recipients. Of course, pharmacokinetics and pharmacodynamics are strictly related to each other, but they are two different aspects of drug therapy.

## 2. The Beginning of Haemophilia Therapy

After the discovery of cryoprecipitate on 1964 by J. Pool [2,3] the lyophilized clotting factor concentrates become available at the beginning of ’70 [4]. The first PK studies were limited to the evaluation of percentage In Vivo Recovery (IVR) and Half-life (HL), by means of the graphic method [5,6]. According to the formula developed by I.M. Nilsson in 1977 [7], percentage IVR considers the dose of FVIII administered and the plasma volume, evaluated by haematocrit. Apart from the inaccuracy of evaluating plasma volume by means of haematocrit, because of the plasma trapped within red cells, the assumption that Volume of distribution (Vd) of FVIII is limited to the plasma pool is inaccurate. The formula proposed by Prowse [8] is a more robust evaluation of incremental IVR because it is a very simple ratio between the post-infusion peak (IU/dL) and the administered dose of FVIII/IX (IU/Kg). Unfortunately, it is difficult to predict the time when the peak will occur and this is the reason why some PK protocols recommend three samples at 15, 30, and 60 min after the end of infusion to pick up the peak and to have a real evaluation of IVR, according to the post-infusion peak. In some cases, the incremental IVR has been calculated based on maximal FVIII/IX concentration at time 0, the extrapolated Cmax, being in this case IVR the ratio between Cmax and dose, a parameter usually provided by the software. If the dosage has been entered as dose/kg (IU/kg), the ratio Cmax/Dose is equivalent to incremental IVR, expressed as IU/dL/IU/Kg. The complete unreliability of IVR to tailor replacement therapy of haemophilia was proved by S. Bjorkman [9]. Even though the intra-patient variability of IVR was less than that of the inter-patients, the role of this parameter might be limited to on-demand single dose therapy. IVR can hardly be used for individualising therapy of haemophilia patients on prophylaxis.

## 3. Plasma-Derived Clotting Factor Concentrates and Their Pharmacokinetics

At the beginning of 1980, cryoprecipitate by single donor was still used in some haemophilia A patients because of fear of hepatitis virus contamination of commercial, large pool, FVIII concentrates after their introduction on 1972 in clinical practice [10,11]. At the Haemophilia Centre of the University Hospital of Florence, Italy, we conducted a study to compare the in vitro characteristics and PK of cryoprecipitate and one intermediate purity and two high purity FVIII concentrates in a small cohort of patients (*n* = 28). For the first time, a two-compartment model analysis was used to evaluate the PK of FVIII products. The study showed that HL was longer in patients treated with cryoprecipitate and intermediate purity FVIII concentrate, 15.63 ± 4.75 h and 13.62 ± 4.01 h respectively, than in patients treated with the two high purity FVIII concentrates, 10.40 ± 2.47 h and 10.94 ± 8.01 h, respectively [12]. Since the first experiences of PK, we realised the occurrence of a very large inter-patient variability of PK outcomes when the patients were treated with different clotting factor concentrates [13]. Anyway, the inter-occasions variability of each patient, when treated with the same concentrate, was lower [14]. This was a prerequisite for developing new strategy for treatment of haemophilia patients based on PK. At that time, the dosing of patients was calculated based on IVR [15] considering the body weight and fluid of each patient. The need of maintaining a safe haemostatic level and the high cost of clotting factor concentrates prompted us to apply to haemophilia treatment the methods of individualization of drugs with narrow therapeutic index. As a matter of fact, the clotting factor concentrates do not show exactly this characteristic because the lower level of haemostatic efficacy depends on the intensity of bleeding, spontaneous or traumatic. It is claimed that patients with severe haemophilia (FVIII/IX < 1 IU/dL) should be maintained at 12–15 IU/dL to completely avoid any bleeding [16]. As far as the upper limit is concerned, this could be placed at any higher level between 15 and 100 IU/dL without any adverse event. Regardless, there is a need to tailor the replacement therapy according to the different clinical situations, avoiding the wasting of very expensive drugs. We applied to the haemophilia treatment the method of Sawchuk-Zaske [17] based on the administration of a test dose followed by limited plasma sampling [18,19]. The serial FVIII concentration-time data were used to calculate individualized estimates of PK and then the dosing of each patient. Only three post-infusion samples were needed to be fitted to the one-compartment model to estimate the HL, elimination rate constant K 1-0 and Vd. At the end, these parameters were used to determine the most suitable dosage for the patient. The difference between observed and predicted factor concentrations, i.e., the sum of squared residuals (SSR), is considered as an index of goodness of the method. A hand-held calculator program was written by our group in 1984 [20], validated subsequently [21] for individualization of haemophilia therapy. The Clearance (Cl), i.e., the amount of drug removed from the plasma during the post-infusion time of the administered factor evaluated by single dose PK, is the more accurate outcome to predict the right repeated dosing of each patient. Unfortunately, a well-designed PK is very demanding for both treatment providers/nurses and patients. The need to collect at least five or six blood samples during the two or three days after the test dose infusion may be difficult to be satisfied because of the logistic organization. Venous access problems in children or travel difficulties for patients living far from the Haemophilia Centre represent the major obstacle to make the PK well accepted by patients and their parents. To overcome these difficulties in forecasting the more appropriate replacement therapy in haemophiliacs, the Bayesian method was implemented in a simple software running on the hand-held Hewlett-Packard HP-41 calculator, based on the one-compartment model [22,23,24]. This method, used some years before for other drugs [18,19] is based on the comparison of the outcomes of some PKs performed in a large cohort of patients, the apriori population data, with the single patient data, a-posteriori data, to predict a dosage regimen. According to this Bayesian compromise, the individual PK parameters are compared with the population PK (PopPK) parameters, derived from previously PKs. The prerequisite of PopPK is the availability of a large body of apriori data, but even sparse data can be cumulated in the population. Few, two or four drug concentration values, may allow the estimation of individual parameters, such as Cl, HL, and Vd, to be compared with population parameters to achieve the compromise. When few drug’s concentrations from the patient are available, the parameters resulting from Bayesian compromise will be shifted toward the values derived from a large population and vice versa when several patients’ data are available and the size of the population is small. In 1985, we applied the model-independent method, also called Non-Compartment Analysis (NCA), to FVIII concentrates kinetic evaluation [25]. This method does not require the assumption of a specific model. FVIII or FIX biological pathways and multi-compartmental distribution are not very well defined, and monophasic or biphasic linear decay can not a priori be forecasted. As far as FVIII is concerned, intermediate purity plasma-derived concentrates display more frequently a monophasic decay while that of high purity ones and recombinant concentrates is frequently biphasic (Table 1). These data have been confirmed in the cross over PK study comparing pdFVIII and rFVIII concentrates PK in a multicentre study. The difference observed in this study was limited to the Alfa distribution phase, showing the rFVIII concentrates a steeper initial decay than that of pd-FVIII concentrate [26]. Probably the FVIII/vWF complex present in the final formulation of pdFVIII concentrates allowed their longer permanence in central plasma compartment. NCA shows to be very robust from a statistical point of view and it does not need a best fitting procedure of data points. The Area Under the Curve (AUC) is calculated according to the trapezoidal rule, a broken curve connecting the concentration/time points. This method is particularly recommended for comparison of two different concentrates in the head-to-head studies, if the samples’ design is the same for both concentrates. The terminal HL, based on the last points of the decay curve, may vary according to the number of best-aligned points included in the calculation by the user or by the software: the last being the most accurate choice! The model-independent method offers a limited view of biological phenomena with comparison to compartmental methods. These are considered much more creative tools to describe the drugs’ in vivo behaviour. According to the opinion of Gabrielson J. and Weiner D., “*The use of so-called model independent descriptions of drugs may be likened to the limited view of a person who gathers flowers and describes them with beautiful pictures. The flowers may even be pressed and preserved in a book to show that they really exist.*” “*A more productive method of learning about flowers is to gather the seeds and plant them. The growth of the flower can be studied in all its stages and cross-pollination used to develop stronger and more beautiful hybrids.*” [27]. For sure, compartment analysis can adhere better to complex, dynamic, clotting factors behaviour in recipients, if we have enough knowledge of them, as derived from available physiology and biochemistry studies. Vice versa, fitting different models to data can help us to understand the kinetics and the efficacy of the drugs. One- and two-compartment models have been fitted to the FVIII, two- and three-compartment to FIX concentration/time data. One-compartment model fits very well a monophasic decay when the concentration/time function can be explained by only one exponential formula *C*_(*t*)_ = *D*/*V*·e^−*k*·*t*^. Monophasic decay was observed very frequently (87%–56%) in intermediate purity concentrates [13], while biphasic decay was more frequent (60%–93%) in monoclonal or high-purity pd-FVIII and recombinant FVIII concentrates (unpublished data) (Table 1). The concentration/time function of biphasic decay is explained by the following formula, where both the distribution Alpha and elimination Beta phases are considered: *C*_(*t*)_ = *A*·e^−α*t*^ + *B*·e^−β*t*^. The more appropriate model, one- or two-compartment, should be judged by statistical parameters, as the Sum of Squared Residuals (SSR), being the residuals the difference between the observed and predicted, by the model, concentration/time points, or Akaike Information Criterion (AIC) or Schwarz Bayesian Criterion (SBC). The model associated with the lower SSR, AIC, and SBC is regarded as the one best fitting the data. Both the Model-independent method and one compartment method were used to analyse 86 single-dose pdFVIII curves obtained in 56 haemophilia A patients and 47 repeated doses obtained from 32 haemophilia A patients [14]. Multivariate analysis of single-dose PK outcomes revealed a weak correlation between Cl and doses, a bit stronger between Volume of distribution according to the terminal half-life (Vz) and Doses, but no significant relationship during repeated doses regimen was revealed. The overall intra-patient coefficient of variation (CV) was ranging from 20.7% to 23.2%. Many factors can explain this variability: (1) errors of FVIII laboratory assay, (2) discrepancy between the true FVIII potency and the labelled potency of concentrate, (3) discrepancy between the FVIII observed decay and the model-predicted shape, and (4) the true intra-individual variability.

Apart from the large inter-patients variability of PK among non-bleeding patients, surgery may add another cause of variability due to the clotting factors consumption and the bleeding in the perioperative period. This topic has been addressed by a multicentre study on FVIII PK during surgery [20] in a cohort of 20 haemophilia A patients. Ten patients underwent a pre-surgery single dose PK study. Everyone received a loading dose before surgery and repeated-dose treatment during the post-operative period. Several intermediate or trough FVIII assays were used to build the decay curves, analysed by constant- or variable-elimination, multiple-dose one-compartment model. In 50% of patients, the FVIII elimination resulted increased (HL 9.6 h), regardless of the type of surgery. A nomogram, based on “single point after a single dose” method was developed by means of 20 patients PK data, to predict the replacement regimen for a desired steady-state of FVIII concentrations, 30, 60, or 90 IU/dL.

The introduction of new pdFVIII concentrates, purified by means of monoclonal antibodies, prompted us to evaluate their PK characteristics. Immunoaffinity chromatography with anti-FVIII Mab was used to producing Hemofil M (Baxter) solvent/detergent treated, and anti-vWF Mab to produce Monoclate P pasteurised and Monoclate HT heat treated. The three products were tested in 10 patients with haemophilia A using the same dosing and sampling design. The PK analysis, performed with one- or two-compartment analysis, each selected according to the lower SSR, and non-compartmental method, did not show any significant difference among the three monoclonal concentrates in term of Cl, mean residence time and Vd [28]. More interesting was the PK study performed in 11 vWD type III patients after single dose infusion of Hemofil M and of Recombinate in two others [29]. The vWF/FVIII ratio was 0.02 in Hemofil M, while Recombinate was completely free of vWF. The HL of FVIII was very short, about 2.8 h in patients treated with Hemofil M and 4.5–4.7 h in patients treated with Recombinate. Similar findings have been reported by Lethagen S. [30]. These observations, compared with the previous study on Mab purified FVIII concentrates in haemophilia A patients, highlighted the very important role of vWF of recipients as the carrier of infused Mab purified or recombinant FVIII concentrates. As a matter of fact, the binding of infused FVIII to vWF is very fast, within few seconds [31], and 90% of infused 125-Jodine FVIII can be recovered from the recipients’ plasma by cryoprecipitation [32].

## 4. DNA-Recombinant Clotting Factor Concentrates and Their Pharmacokinetics

The first recommendations for clotting factor PK were released in 1991 on behalf of Sub-Committee on Factor VIII and Factor IX of the Scientific and Standardization Committee of ISTH and were subsequently updated 10 years later [33,34]. The optimal design of cross-over studies to prove the bioequivalence of new recombinant concentrates was defined in terms of samples’ size and timing, outcome variables both by compartmental methods and model-independent analysis, single dose’s amount, and analytical methodology (reference standards, reagents and instrumentation). The well-known variability of FVIII assay [35,36,37,38] had jeopardised the determination of IVR and reliability of post-infusion FVIII/IX concentrations [39]. A European multicentre study aimed to assess the reproducibility of one- and two-stage clotting methods and chromogenic assay of FVIII showed a good agreement between one-stage clotting and chromogenic assay in a wide range of concentrations, while two-stage clotting assay was not well reproducible [40]. The same PK study design was employed for another multicentre study comparing two pasteurised clotting factor concentrates, Haemate P vs. FVIII CS from CSL Behring [41]. Similar discrepancies between one-stage clotting and chromogenic assay resulted some years later [42] in a multicentre PK study of the new B-domain deleted FVIII concentrate, moroctocog alfa. FVIII concentrations ranging 0.25–1.00 IU/mL assayed by chromogenic method against a plasma reference standard resulted 20% higher than those assayed by one-stage clotting method. FVIII concentrations ranging 0.25–0.01 IU/mL resulted about 15% higher when assayed by one stage clotting method. This paper deserved also a commentary by P. Lollar [43] which emphasized the difficulties and variability of the biological assay of FVIII. The first PK study on new recombinant FVIII concentrate, Recombinate (Baxter) was performed to compare the decay of this product with Hemofil M [44] in the frame of Recombinate Study Group. The cross-over study, conducted in 47 haemophilia A patients, showed higher HL and smaller Cl of Recombinate. The more accurate FVIII assay can be achieved using haemophilia A plasma as diluent instead of buffer [45]. Also, Prothrombin Complex Concentrates (PCC) and high-purity Factor IX concentrates have been submitted to PK analysis at the beginning of 1990 [46,47]. A review of PK outcomes of PCC has been presented at ISTH congress of 2007 in Geneva, as reported in Table 2 [48]. A very large Vd was common to all PCC, except the four-factor concentrates and intermediate purity Bebulin.

In 1992, PK of clotting factor concentrates started to be investigated in Sweden. In his first paper, S. Bjorkman emphasised the misleading nature of percentage IVR and the need for accurate evaluation of potency of the final formulation of concentrates [52]. The poor reliability of incremental IVR for dosing haemophilia patients was again and better defined some years later [9]. Prophylaxis was very popular in Sweden after the first experience of I.M. Nilsson [53] when PK was applied to tailor prophylaxis for the first time in that country [54,55,56]. PK driven prophylaxis of haemophilia A patients resulted as more cost-effective: a PK-tailored two-day regimen reduced the average FVIII consumption by 43% and the daily dose by 82%. Of course, this regimen also reduced the quality of life of patients due to the frequent venous access [57]. Similar findings derived from a PK driven prophylaxis in haemophilia B [56]. The definition of plasma samples’ timing was investigated in six patients infused with a pdFIX concentrate: in this study [58], the optimal time was 56 h post-infusion because after this time limit the PK outcomes remained unchanged. It is well known that the gold rule to achieve a complete and accurate definition of drug PK is to prolong the blood samples’ collection until the baseline drug concentration has been achieved. Of course, the amount of drug infused determines the proper time of blood collection. In this study [58], two-compartment model fitted the FIX disposition, *t*_1/2_ beta 34 h, MRT 37 h, Cl 4.0 mL/h/kg and *V*ss 150 mL/kg. This last outcome showed that about 44% of infused FIX spends its MRT in the extravascular compartment, after diffusion in the extracellular and lymph fluid. The binding of FIX to endothelium [59] and to collagen type IV [60] may explain its very large apparent Vd, larger than that of FVIII. Notwithstanding this large Vd, the half-life of FIX is quite longer than that of FVIII while the Cl of both factors is very similar: the flow back of FIX from extravascular compartment to plasma compartment may explain these peculiar characteristics of FIX PK [61].

When recombinant FIX concentrate (rFIX) became available, the phase I/II PK study was limited to IVR (0.84 IU/dL per IU/kg) and HL (18.10 ± 5.10 h) [62,63]. Similar findings but again limited to IVR and HL were reported some years later in two other independent studies [64,65]. The relationship between FIX:Ag and IVR was investigated in both these studies and IVR resulted lower in haemophilia B patients Cross Reacting Material (CRM) negative. When the production of FIX:Ag is low, the extravascular space is partially empty and the infused FIX may flow out of plasma compartment. In the same period, the data of 56 haemophilia B patients treated with Benefix during one of the previous studies [64] were analysed by Bjorkman [66] and, for the first time, also Cl and Vd were reported. The absolute values of these primary PK parameters resulted increasing with the weight and age of patients during the childhood and adolescence, reaching a plateau during the adulthood. But Cl and Vss, when corrected for body weight, showed an inverse relationship with the age. So, the Cl resulted in 10.4 ± 2.25 mL/h/kg and the Vss 270 ± 70 mL/kg in patients aged 4–9 years but 7.15 ± 1.39 mL/h/kg and 190 ± 40 in patients aged 30–39 years, respectively. FVIII and FIX undergo different changes of their in vivo behaviour during the patients’ life. The absolute Cl (mL/h) of both factors show a decrease, more evident for FIX, inversely related to the increase of age and body weight of patients growing from childhood to adolescence [61]. The change of Vd of FIX is parallel to the decrease of Cl but that of FVIII is quite stable during all life of patients. Because the half-life is proportional to Vd and inversely related to Cl, the parallel change of Cl and Vss of FIX makes the changes of HL very trivial during the growth of haemophilia B patients. On the contrary, the decrease of FVIII Cl in adolescent or adult patients, while the Vd or Vss are stable, causes a significant improvement of FVIII HL in haemophilia A patients during their growth. The small changes of FVIII Vd is a consequence of FVIII binding to vWF which limits the effusion of this factor into the extravascular space, making the change of Cl more effective on its HL [61].

After the first release of FVIII/FIX SSC recommendations for PK issued on 1991 [33], a second release was published on 2001 [34] where some important features of single dose PK were addressed. The size of studies was defined according to the following formula [67]:

*n* ≥ (*t*[*a*/2, 2*n* − 2] + *t*[*b*, 2*n* − 2])2 × (*cv*/20)^2^

where *t* is the Student’s *t* test coefficient, a and b are the significance levels of type 1 error (0.05) and type 2 error (0.20) respectively, and CV is the coefficient of variation (generally 30%). According to these values, the minimal size of a PK cross over bioequivalence study should be 40 patients, treated with both products. To have a good description of PK, the following parameters were indicated: AUC, AUMC, HL, Incremental IVR, Mean Residence Time, Cl, Vd at steady state, Cmax, and Tmax. The wash out period is generally defined as five times the HL. The suggested time’s points for patient sampling were the following: baseline, 0.25, 0.50, 1, 3, 6, 9, 24, 28, 32 post-infusion hours for FVIII, and another point at 72 h for FIX. The suggested dose was 50 IU/kg and 75 IU/kg for FVIII and FIX respectively. This approach was regarded as very much demanding first for haemophilia children [68] and several attempts were done to decrease the number of points. Unfortunately, both compartmental and non-compartmental methods require a minimal number of points to achieve a good description of factor concentrates distribution in the recipients’ body. The broken line connecting the FVIII/times points of NCA may overestimate the AUC. In particularly, the points around the “knee” of the biphasic curve, generally occurring between the 7th and 9th hour, are of crucial importance to avoid a large overestimation of the curve. Compartmental methods also need many points to minimise the residuals, i.e., the discrepancy between observed and predicted FVIII/IX concentrations [69]. SSR, better if derived from weighted data (SWSR), AIC and SBC should be considered to validate the best model to fit a single dose PK. A reduced number of well-spaced samples (baseline, and 1, 7–9, 24, 48, 72 post-infusion hours) has been recommended to limit the error of Cl to 5% [66] when calculated with by NCA and the error of the central volume to 1% by compartmental analysis [70]. In particularly, no improvement of the PK outcomes was observed by increasing to six the number of points for FVIII biphasic decay [70]. If the minimal concentration/time point is five for a biphasic FVIII decay curve, the right number for a good analysis of a FIX PK is at least six or seven. The same topic was addressed by the analysis of a large body of data, derived from three phase I/II octocog alfa PK studies conducted in 52 1–6-year old and in 100 10–65-year old haemophilia A patients [71]. About 50% of total inter-patient variability was due to the reduced blood sampling schedule adopted for children. The intra-patient variability in the 10–65-year old patients was smaller than that of the group of younger patients. The conclusion was that different blood sampling schedules do account for the differences in the outcomes of PK studies conducted with different designs. Of course, when the difference between two concentrates is big, as for instance between the current rFIX and rIX-FP or N9-GP, is not easy to adopt the same design for both concentrates. The gold rule of pharmacokinetics (that each PK should be prolonged until the drug baseline has been achieved again) does not necessarily require the same sampling design, but that is the only way to have an accurate and true description of the drug in the body. In all the phase I/II of new extended HL rFIX concentrates [72,73], the pdFIX and rFIX single dose have been stopped too early, at 48 h, when the FIX level was till high. Only in phase 3 head-to-head study of rFIX-Fc, the PK of pdFIX and rFIX have been prolonged up to 72 and 96 h [74]. It is easy to understand how prolonged sample timing causes a larger AUC, a smaller Cl, and a longer HL, as shown by a recent Italian multicentre PK study [75]. Similar findings have been observed in a PK study of nonacog alfa conducted in China [76]. Another intriguing issue of pharmacokinetics is the problem of the high baseline value, frequently due to a short wash out, not always less than five times the HL of the drug under investigation. The endogenous FVIII/IX level should be subtracted from all post-infusion points, as suggested by Messori [77]. On the contrary, the residual FVIII/IX present at baseline from the previous infusion will undergo a similar decay as the infused concentrate. Post-infusion FVIII/IX concentrations should be adjusted according to the proportion of baseline to maximum peak; the formula proposed by Bjorkman [71] is the following:

Adjusted FVIII/IX = measured level × (1 − (baseline level/peak level)]

If a well-designed single dose PK is considered too demanding, especially for children, at least regular controls of trough and peak during prophylaxis or the periodical check-up at haemophilia centre must be recommended. The frequency of breakthrough bleedings during prophylaxis has been related to the time the patients spent below the 1% trough [78] and a complete bleeding free life seems to be guaranteed by 12%–15% trough [16]. The decay of FVIII and especially that of FIX is biphasic and it is easy to understand as two concentrates with the same peak and the same trough may have a different AUC according to the more or less biphasic decay. AUC, the more robust outcome of PK, depends on the time and the quantity to whom the patient has been exposed. The role of peak, AUC and trough in the prediction of clinical outcome of every-third-day prophylaxis has been recently very well emphasised [79]. A very high FVIII trough, >27 IU/dL prevented completely the spontaneous bleedings [79]. Cl, the amount of drug removed from plasma pool during the time, is derived by the ratio Dose/AUC and summarizes very well the meaning of peak, AUC and trough. Although on-demand therapy can be approximately dosed based on IVR, the dose and interval (Tau) of repeated bolus administrations or continuous infusion can be derived from the following formula:

Dose (IU/kg) = Clearance (dL/h/kg) × Desired plasma level (IU/dL) × Tau (h)

From which it is easy to understand that the continuous infusion rate is the cheaper way of replacement therapy while larger the interval between bolus, higher is the dose.

## 5. Population Pharmacokinetics

The wide inter-patient variability of Cl among the haemophilia patients together with the smaller intra-patient or intra-occasion variability prompted us to apply for the first time the population PK (PopPK) to the haemophilia therapy and develop a calculator program for the Bayesian method [23,80]. “One size does not fit all” recently became a very popular slogan, and after our pioneering paper, several attempts have been made to apply PopPK to clotting factor concentrates. Really, PopPK has been developed for drugs with narrow therapeutic index, that is not exactly the CFCs characteristic. The minimal haemostatic level of FVIII/IX may vary according to the clinical situation from 2 IU/dL to 15–20 IU/dL but the range of the upper limit is larger, 60–160 IU/dL! Of course, PopPK may make the replacement therapy not only safer but first may improve its cost/effectiveness. The major advantage of PopPK is that even sparse samples from all individuals can be combined to build the model. On the contrary, the traditional Standard Two-Steps (STS) method [81] is based on (first step) the estimation of PK parameters of each patient submitted to a lot of blood samples and on (second step) the statistical and regression analysis to investigate the effects of patients’ covariates. After the definition of the model by PopPK, only a few samples, 2–3 well spaced, can be used to check if a new patient’s decay fulfils with the model. Two software packages are so far available for individualization of haemophilia therapy by means of PopPK, both based on NONMEM procedure: MyPKFit (Baxalta, part of Shire) and WAPPS [82]. The a priori data of MyPKFit have been derived from merging three PK phase I/II studies of Advate, 100 conducted in adolescent-adult patients and 52 in children. The PK data of the total population used in MyPKFit were very spread out: IVR was ranging from 1 to 5 IU/dL/IU/kg and the decay curves were biphasic. This large variability was due to the merging of 2 adults and 1 children PK studies, all together. Only two points, at 3rd and 24th hour, are enough to build the decay curve of a single patient, to check by MyPKFit if it is within the 95% confidence limit of average decay curve. Because the aim of PopPK is to better tailor the therapy of one patient according to the population model, an appropriate validation procedure must assess the outcomes during the follow-up: changes of bleeding rate, clotting factor consumption, quality of life of patients, side effects must be accurately recorded for each patient after a change of prophylaxis regimen. Negative outliers with respect to the average decay curve should increase their dosage or reduce the interval: this change is generally the best accepted by patients. On the contrary, the positive outliers should decrease the dosage or increase the interval: a choice not always well accepted by the patient and the doctor! A PopPK 2-compartment model of rFVIII concentrate was developed by Bjorkman [83] from the 236 PK of 152 patients to investigate the relationship between the age and body weight. Afterwards, a PopPK 3-compartmet model of rFIX was developed [84] for tailoring prophylaxis in haemophilia B patients according to their FIX trough level. Also, a PopPK 3-compartment model of high-purity and monoclonal purified pdFIX concentrates was developed, merging the data of 5 different single dose PK studies conducted in a small cohort of patients [85]. Based on FVIII 3 or 10 IU/dL trough [86] needed to avoid bleedings, two PopPK models were developed for full-length and rFVIII-Fc. According to this model and the covariates taken from the published clinical trials, a simulation in 1000 patient populations submitted to different prophylactic regimens was developed. Simulated FVIII level remained >3 IU/dL in 57% of patients treated every 48 h with 30 IU/kg of rAHF-PFM compared to 41.1% of patients treated with the same dose of rFVIII-Fc every 72 h [86]. Even though the different assumed covariates of the two trials (body weight and age for rAHF-PFM and VWF, body weight, and haematocrit for rFVIIIFc), the difference between the two prophylactic regimens was not substantial. On the contrary, the decrease of a few annual venepunctures (182/year for Advate, 120/year for rFVIIIFc) may be very relevant for haemophilia children on prophylaxis [87]. Recently the PK parameters were used to simulate the decay curves in thousands of patients! This modelling is based on the very popular software Markov Chain Monte Carlo, a Bayesian method that evaluates the likelihood function of events starting from parameters previously achieved [88]. This procedure can be very useful but also affected by some biases when the comparison between two products is not based on the exactly same experimental conditions. Furthermore, it is wrong to predict the incidence of clinical events, like annual, or worse “annualised”, bleeding rate (ABR) or other clinical endpoints, based on what happened in the starting clinical trial of the new drug. We must keep in mind, that PK is a surrogate of efficacy and that other factors (lifestyle, trauma, joint inflammatory conditions, muscle tone, etc.) above CFC level may determine the bleeding in haemophilia patients.

## 6. Conclusions

During the last 30 years, pharmacokinetics has become a very popular tool to study the in vivo behaviour of CFCs. Considering that about 20 new FVIII and five FIX concentrates became available in this period, their bioequivalence or characteristics have been studied by means of current PK methods. Even though PK meaning is different from pharmacodynamics and is only a surrogate of efficacy, the relationship between the plasma level of deficient factor and bleeding makes the PK a useful tool to roughly predict the outcome of replacement therapy. Unfortunately, bleeding is a multifactorial event, and the efficacy of CFCs must be proved in vivo. Never the less, excellent results have been achieved by tailoring prophylaxis of haemophilia patients according to their individual PK outcomes. The implementation of extended half-life (EHL) CFCs in our clinical practice made the PK a very useful tool to confirm in switched patients the PK parameters achieved during phase I/II studies. “One size does not fit all”: the behaviour of the same CFC may be different in each patient. This is the reason why a single dose PK has been recommended by UKHCDO for previously treated patients when switched from current CFCs to a new EHL FVIII/IX concentrate as well as for untreated ones at their first treatment [89].

## Figures and Tables

**Table 1:** Different prevalence of monophasic or biphasic decay in intermediate or high purity FVIII concentrates. *N*: number of PKs.

Product	*N*	Formulation	Monophasic	Biphasic
Kryobulin	39	Intermediate Purity	34 (87%)	5 (13%)
Kryobulin TIM3	17	Intermediate Purity	12 (73%)	5 (27%)
Haemate P	72	Intermediate Purity	40 (56%)	32 (44%)
Monoclate HT	10	Mab purified	4 (40%)	6 (60%)
Monoclate P	10	Mab purified	3 (30%)	7 (70%)
Recombinate	43	rDNA derived	10 (23%)	33 (77%)
Kogenate	27	rDNA derived	3 (11%)	24 (89%)
Hemofil M	43	Mab purified	3 (7%)	40 (93%)
Koate HS	14	Mab purified	1 (7%)	13 (93%)

**Table 2:** A summary of outcomes of some PK studies om pd-FIX concentrates. The low IVR and the huge Volume of distribution of about all products are remarkable, in contrast with a quite long MRT or Half-life.

Product	Clearance (mL/h/kg)	MRT (h)	Half-Life (h)	Volume of Distribution (mL/kg)	IVR (%)	Reference
Bebulin	4.99 ± 2.01	22.9 ± 10.6	15.87 ± 7.35	99.9 ± 35.5	59.8 ± 16.9	[49]
Preconativ	4.78 ± 2.63	24.5 ± 8.6	16.98 ± 8.6	127.01 ± 60.8	54.0 ± 30.8	[46]
Immunine	8.89 ± 2.91	23.86 ± 5.09	16.53 ± 3.53	204.56 ± 55.91	41.54 ± 12.94	[47]
Replinine	3.08 ± 0.83	55.8 ± 19.5	38.67 ± 13.51	165.5 ± 55.4	86.21 ± 6.4	[50]
FIX-SD	7.4 ± 0.8	45.6 ± 4.5	31.60 ± 3.12	162.9 ± 47.8	40.4 ± 8.9	[51]
FIX-SD Nanofiltered	6.9 ± 1.2	44.2 ± 4.9	30.63 ± 3.40	155.4 ± 46.4	47.0 ± 8.9	
